# Whole-Genome Sequence of Oryctes rhinoceros Nudivirus from Riau Province, Indonesia

**DOI:** 10.1128/MRA.01476-20

**Published:** 2021-12-02

**Authors:** Yudistira Wahyu Kurnia, Zulfikar Achmad Tanjung, Condro Utomo, Mohammad Naim, Elizabeth Caroline Situmorang, Tony Liwang

**Affiliations:** a Plant Production and Biotechnology Division, PT SMART Tbk, Bogor, Indonesia; b SMART Research Institute (SMARTRI), PT SMART Tbk, Riau, Indonesia; KU Leuven

## Abstract

A double-stranded DNA virus, Oryctes rhinoceros nudivirus (OrNV), was detected in the total DNA of diseased larvae of *O. rhinoceros* in Riau Province, Indonesia. The complete genome sequence was 124,926 bp long and encodes 123 open reading frames (ORFs). This strain belongs to the family *Nudiviridae* and was designated LiboV.

## ANNOUNCEMENT

Oryctes rhinoceros nudivirus (OrNV) has been used for decades as an effective biocontrol agent against the coconut rhinoceros beetle (CRB; Oryctes rhinoceros), a major pest of the oil palm (1–4). However, the emergence of OrNV-tolerant CRB has been recently reported (5). The genomic characterization of OrNV from several geographic areas using complete genome sequencing is hence important (6, 7). To date, only two OrNV full genome sequences are available, isolate Ma07 from Malaysia (8) (the first one described) and isolate Solomon (SI) from the Solomon Islands (6). Here, we report the whole-genome sequence of an OrNV detected in diseased CRB larvae found on an oil palm estate in Riau, Indonesia (0°55′11.5″N, 101°17′09.4″E).

Initially, 50 g of frozen field-collected diseased CRB larvae showing symptoms of translucent, soft bodies were thawed and powdered using liquid nitrogen. The DNA was then extracted using a DNA miniprep kit (Zymo Research, USA). An amplicon of 945 bp was detected following a PCR amplification of the DNA using OrNV universal primers (9, 10).

Full-genome sequencing of the above-mentioned DNA was conducted at Beijing Novogene Biological Information Technology Co., Ltd. (China). Briefly, the library preparation was conducted using the NEBNext Ultra II DNA library prep kit, and fragmentation was performed using a Covaris ultrasonicator. The fragments were end-polished, A-tailed, and ligated using the full-length adapter for Illumina sequencing, with further PCR amplification. The quality and quantity were assessed using the Agilent 2100 Bioanalyzer. All libraries were sequenced using the Illumina NovaSeq 6000 platform, generating a total of 104,938,415 paired-end 150-bp reads. The raw reads were trimmed using the Trim Ends tool (Geneious 2019.1.1) and were then *de novo* assembled using Megahit (11), which generated 1,845 contigs (range, 200 to 17,139 bp). The contigs were then mapped to the reference isolate Ma07 (GenBank accession no. NC_011588) using Geneious Prime, resulting in 704 hits. Gaps found within the *de novo* contigs were filled with the raw reads using the Geneious alignment algorithm. All tools were run with default parameters unless otherwise specified.

A genomic OrNV DNA sequence (namely, LiboV) of 124,926 bp, with a GC content of 41.70%, was identified, with an average coverage of 1,150×. Pairwise comparison of the LiboV whole-genome sequence to Ma07 and SI (GenBank accession no. MN623374.1) showed 97.46% and 96.73% nucleotide sequence identities, respectively. A total of 820 reads spanning the start and the end of the obtained contigs were identified, suggesting that the genome of LiboV was circular and complete.

The Find ORFs tool (Geneious 2019.1.1) predicted that the LiboV genome encodes 123 open reading frames (ORFs), fewer than previously described for SI or Ma07, which encode 130 and 139 ORFs respectively (6, 8). Sixteen previously annotated hypothetical proteins (6, 8) were not found in the genome of LiboV due to frameshifts. In addition, the rearrangement (inversion) of four genes (*gp131*, *gp132*, *gp133*, and *gp134*) reported in the SI genome when aligned with Ma07 (6) was not found in the current assembly.

Compared to the reference, 213 single-nucleotide polymorphisms (SNPs) were found in 61 genes, which caused 177 amino acid modifications in 32 genes. The OrNV whole-genome sequence from this study provides a valuable resource for future study toward deeper biological and geographical insight into OrNV.

### Data availability.

The whole-genome sequence described here has been deposited in GenBank under accession no. MZ727584. Original sequencing data were deposited in the Sequence Read Archive (SRA), accession no. SRX9638947.

## References

[1] Manjeri G, Muhamad R, Tan SG. 2014. *Oryctes rhinoceros* beetles, an oil palm pest in Malaysia. Annu Res Rev Biol 4:3429–3439. doi:10.9734/ARRB/2014/11023.

[2] Huger AM. 1966. A virus disease of the Indian rhinoceros beetle, *Oryctes rhinoceros* (Linnaeus), caused by a new type of insect virus, Rhabdionvirus *Oryctes* gen. n., sp. n. J Invertebr Pathol 8:38–51. doi:10.1016/0022-2011(66)90101-7. 5905536

[3] Huger AM. 2005. The *Oryctes* virus: its detection, identification, and implementation in biological control of the coconut palm, rhinoceros beetle, *Oryctes rhinoceros* (Coleoptera: Scarabaeidae). J Invertebr Pathol 89:78–84. doi:10.1016/j.jip.2005.02.010. 16039308

[4] Jackson TA. 2009. The use of *Oryctes* virus for control of rhinoceros beetle in the Pacific Islands, p 133–140. *In* Hajek AE, Glare TR, O’Callaghan M (ed), Use of microbes for control and eradication of invasive arthropods. Springer, Dordrecht, Netherlands.

[5] Marshall SD, Moore A, Vaqalo M, Noble A, Jackson TA. 2017. A new haplotype of the coconut rhinoceros beetle, *Oryctes rhinoceros*, has escaped biological control by *Oryctes rhinoceros* nudivirus and is invading Pacific islands. J Invertebr Pathol 149:127–134. doi:10.1016/j.jip.2017.07.006. 28743668

[6] Etebari K, Filipović I, Rašić G, Devine GJ, Tsatsia H, Furlong MJ. 2020. Complete genome sequence of *Oryctes rhinoceros* nudivirus isolated from the coconut rhinoceros beetle in Solomon Islands. Virus Res 278:197864. doi:10.1016/j.virusres.2020.197864. 31945420

[7] Kawakami K, Kurnia YW, Fujita R, Ito T, Isawa H, Asano SI, Binh ND, Bando H. 2016. Characterization of a novel negevirus isolated from Aedes larvae collected in a Subarctic region of Japan. Arch Virol 161:801–809. doi:10.1007/s00705-015-2711-9. 26687585

[8] Wang Y, Kleespies RG, Ramle MB, Jehle JA. 2008. Sequencing of the large dsDNA genome of *Oryctes rhinoceros* nudivirus using multiple displacement amplification of nanogram amounts of virus DNA. J Virol Methods 152:106–108. doi:10.1016/j.jviromet.2008.06.003. 18598718

[9] Richards NK, Glare TR, Aloalií I, Jackson TA. 1999. Primers for the detection of *Oryctes* virus from Scarabaeidae (Coleoptera). Mol Ecol 8:1552–1553. doi:10.1046/j.1365-294x.1999.07072.x. 10617346

[10] Moslim R, Kamarudin N, Ghani IA, Wahid MB, Jackson TA, Tey CC, Ahdly M. 2011. Molecular approaches in the assessment of *Oryctes rhinoceros* virus for the control of rhinoceros beetle in oil palm plantations. J Oil Palm Res 23:1096–1109.

[11] Dinghua L. 2015. MEGAHIT: an ultra-fast single-node solution for large and complex metagenomics assembly via succinct de Bruijn graph. Bioinformatics 31:1674–1676. doi:10.1093/bioinformatics/btv033. 25609793

